# Statistical issues in the analysis of non-pharmacological therapy trials with clustering by care-provider or therapy group

**DOI:** 10.1186/1745-6215-12-S1-A21

**Published:** 2011-12-13

**Authors:** Chris Roberts

**Affiliations:** 1 University of Manchester, Manchester, M13 9PL, UK

## 

In trials of physical and talking therapies or group administered treatments, clustering of patients within care-provider or treatment group has implications for sample size and statistical analysis analogous to those found in cluster randomised trials [1]. Between-cluster variation reduces precision and power when estimating treatment effects. Statistical analyses that fail to take account of this form clustering rest on the assumption of no clustering effect and may therefore lack conclusion validity.

In trials with treatment related clustering, the cluster size may differ systematically between treatment arms, due to differing number of therapists in each arm or differences in the size of therapy groups between arms. The intra-cluster correlation due to therapist or therapy group may also differ between treatment arms. An extreme case of such heterogeneity is the partially nested design in which the clustering effect is absent in one treatment arm. Where both cluster size and the underlying variance components differ between treatments, failure to correctly model heterogeneity can bias estimates of the intra-cluster correlation coefficient and test size [1]. This contrasts with cluster-randomised trials where the distribution of cluster sizes in each treatment arm will be similar due to randomisation making cluster randomised trials robust to heteroscedasticity.

In a cluster randomised trial, it is generally assumed that each subject belongs to just a single unit of randomisation so that the design is hierarchical. In non-pharmacological therapy trials patients may receive treatment from more than one therapist or in more than one therapy group so the multilevel model is no longer strictly hierarchical. Statistical analysis will therefore require simplifying assumptions regarding the pattern of clustering such as use of a primary therapist or primary group for each patient or the application of a multiple membership model [2].

In conclusion, analysis of trials with treatment related clustering may therefore require more complex methods of analysis than cluster randomised trials.
